# 3D Reconstruction of the Blood Supply in an Elephant’s Forefoot Using Fused CT and MRI Sequences

**DOI:** 10.3390/ani13111789

**Published:** 2023-05-28

**Authors:** Örs Petneházy, Shannon Rück, Endre Sós, László Z. Reinitz

**Affiliations:** 1 Somogy County Kaposi Mór Teaching Hospital, Dr. Baka József Diagnostic and Oncoradiological Centre, Guba Sándor utca 40, 7461 Kaposvár, Hungary; 2 Deptartment of Anatomy and Histology, University of Veterinary Medicine Budapest, István utca 2., 1078 Budapest, Hungary; 3 Budapest Zoo & Botanical Garden, Állatkerti krt. 6-12, 1146 Budapest, Hungary

**Keywords:** elephant, forefoot, digits, blood supply, 3D reconstruction, CT

## Abstract

**Simple Summary:**

The weight of an adult African elephant can be over six tons, and even the smaller Asian elephant can weigh over four tons. Their limbs support the body nonstop because elephants rarely lay down as standing up is a difficult and lengthy process. Consequently, even a small injury to the sole, the digits, or the digital cushion can result in a serious abscess or ulcer on the foot, eventually leading to the death of the animal. Surgical solutions are limited due to the lack of anatomical descriptions of the region’s blood supply. The aim of this study was to provide anatomical guidance for the blood supply of the foot. We created a detailed 3D model of both the skeletal and the arterial structures, explaining the blood supply of the digits, the sole, and the digital cushion. This information may be used in the preservation of this endangered species through a better understanding of foot diseases and planning surgeries in clinically affected animals.

**Abstract:**

Being the largest still-living terrestrial mammal on earth, an elephant’s feet play an important role in its health status. The musculoskeletal structures in the forefoot are well described in the literature, but information about vascularization is limited. The novel aim of this work is to provide anatomical guidance to structures found in the forefoot, focusing on the arterial system. Initially, native CT and MRI sequences were taken of the left forefoot of a deceased 6-year-old female Asian elephant; the foot was then filled with an iodine-containing contrast medium through the a. mediana and the CT scans were repeated in the same position. The images obtained were processed with 3D Slicer software for the 3D reconstruction of the bones and arteries. The results clearly showed the palmar blood supply of the forefoot. A so far undescribed vessel was revealed, stemming from the a. metacarpea, supplying the first digit and the digital cushion. The course of the deep palmar arch’s terminal section was also established. This paper provides the first description of the exact disposition of the arteries in the palmar aspect of an elephant’s forefoot and may be used in planning surgeries in clinically affected animals.

## 1. Introduction

For the description of the anatomy of elephants’ feet, the works of Smuts and Bezuidenhout [1,2], Csuti et al. [3], Fowler and Mikota [4], and Mumby et al. [5] were consulted if not indicated otherwise. For those cases where the Latin names were not mentioned in these papers, we used the official names of the homologous structures in household mammals from the Veterinary Anatomical Nomenclature [6].

Elephants’ feet play an important role in their overall health status [7]. In addition to the main tasks in elephant husbandry such as feeding and cleaning, a large amount of time must be dedicated to the soundness of the feet [4,8]. Due to gravity and their extreme size, it takes more effort for the circulation to lift the blood from the limbs back to the heart; that is why while moving, the so-called “digital cushion” (*torus digitalis*) in the elephant’s foot becomes pressurized and acts like a pump forcing the blood to flow upwards [4].

The roundish forefoot of the elephant is semi-digitigrade, carrying 60% of the total body weight. The limb bones are massive and lack a marrow cavity, which is instead replaced with cancellous bone. The radius is reduced, smaller and shorter than the ulna, which leads to the assumption that the ulna is responsible for most of the weight-bearing aspect of the antebrachium. The carpus contains eight bones in total, divided into two rows, with four bones each. In the first row, from medial to lateral, there are the *os carpi radiale*, *os carpi intermedium*, *os carpi ulnare*, and the *os carpi accessorium*. The second row consists of *os carpale I*, *II*, *III*, and *IV* (C-I to C-IV) which articulate with their corresponding metacarpal bones (MC-I to MC-IV), with the *os carpale IV* also having an articulation with the *os metacarpale V* (MC-V). 

The synovial layer of the carpal joint inserts on every row of the carpus, fully separating the three carpal joints (*articulatio antebrachio-carpea, art. mediocarpea*, and *art. carpometacarpeae*). In comparison to other ungulates, the carpus of elephants allows only little abduction.

There are five metacarpal bones, with the third being the largest, followed by the fourth, second, and fifth, with the first being the smallest [9]. This arrangement of sizes corresponds with the size of the digits themselves as well. Palmar to the *articulatio metacarpophalangea,* each metacarpal bone, except for the first, presents a pair of sesamoid bones. 

Although not externally identifiable, the forelimb consists of five digits (D-I to D-V). For some of the digits, depending on individuals, protective but nonweight-bearing toenails are visible on the dorsolateral surface of the foot. In the majority of Asian elephants, five toenails are present in each forefoot. Sometimes, individual deviations can be seen, bearing fewer toenails than expected. The digits II, III, and IV each have three phalanges. The distal phalanx has a unique spindle-shaped form, and it may not articulate with the intermediate phalanx. It is embedded in soft tissue and attached to a toenail. The D-I bears two phalanges and one single sesamoid bone; D-V has two phalanges. D-I and D-V also stand almost perpendicular to the ground, whereas D-II to D-IV incline in an oblique fashion.

The forefoot features a unique characteristic in elephants, the so-called prepollux or prepollex [3,4,10,11,12]; it is a cone-shaped, elongated rod in the forefoot which is slightly curved and ends bluntly. The prepollex extends distally from *os carpale I* and attaches to the sole, medial to the midline of the foot. These so-called predigits are assumed to act as real “sixth” digits for further stabilization of the carpal and tarsal joints and redirect some of the pressure load from the digits to the aforementioned joints; they are usually of a cartilaginous structure and can become partly or completely ossified over time.

Small individual differences in the skeleton of the forefoot are not rare in elephants, where multipartite, missing, or misshapen phalanges, as well as multiple variations of the proximal sesamoid bones (fused together, separated, or completely missing), can be frequent findings in clinical radiographs or CT scans [8]. 

The blood supply distal to the elbow joint is provided by the median artery (*a. mediana*) which runs along with the median vein and nerve on the medial aspect of the *antebrachium* down to the carpus; from that point on, it becomes the metacarpal artery (*a. metacarpea*). A branch of the median artery in the proximal third of the ulna is the interosseous artery (*a. interossea*), descending to the lateral side of MC-V, and continuing as the fifth digital artery. This vessel is homologous with the common interosseous artery (*a. interossea communis*) of the household mammals.

The metacarpal artery flows deep to the metacarpal bones, giving off the first dorsal digital artery (*a. digitalis dorsalis I.*) before it curves ventrolaterally forming the deep palmar arch (*arcus palmaris profundus*) on the palmar aspect of the digits. From there, metacarpal arteries arise for digits I to V (*aa. metacarpeae dorsales I–V.*); these become palmar digital arteries (*aa. digitales palmares I–V.*), the recurrent branches supplying the carpal joints and the dorsal metacarpal arteries for D-II to D-V, which continue further on as the dorsal digital arteries (*aa. digitales dorsales communes I–V.*). Arteries or branches of arteries that supply the digital cushion (*torus digitalis*) are not explicitly mentioned in the literature. In one work of Weissengruber [12] a band of vessels and nerves is mentioned to be in the sagittal plane of the foot’s palmar aspect in African elephants. In household ungulates, where the digital cushion also has a huge significance in the dynamics of the limbs, two vessels are found to supply it. In horses, this is the *r. tori digitalis lateralis et medialis*, stemming from the *a. digitalis* [*palmaris propria III et IV*] *lateralis et medialis*, respectively. In bovines, the two digits each have a *ramus tori digitalis* stemming from the *a. digitalis [palmaris propria III et IV] axialis et abaxialis*, respectively. 

The nervous system of the elephants is also not widely studied [13], but the innervation of the forefoot digits, and the corresponding branches of both the *n. medianus*, *n. radialis*, and *n. ulnaris*, are well explored. In contrast, the nervous supply of the digital cushion is not explained in any study. There is also no detailed information provided about the joints, tendon sheets, and possible connections between the joint cavities other than Weissengruber’s work on the stifle [14].

Due to the lack of a comprehensive anatomical description of the region, case reports and clinical studies are not unequivocal when explaining the given procedure, therefore they are difficult to duplicate [15,16]. During surgical procedures, the approach and exploration of the region are occasional, mostly based on the visible superficial structures [17]. One study reported that the distribution and size of the vessels were unexpected during the removal of a digit, which made the procedure more difficult [15]. The effect of the antibiotic therapy applied in osteitis and osteomyelitis can be enhanced with local intravenous administration, which is currently executed with an ultrasound-guided search of a superficial vessel, without understanding its connection to the systematic circulation [18].

Anatomical descriptions of elephants are often based on standard anatomical and histological techniques, focusing on microanatomy and biomechanics together with musculoskeletal anatomy [19,20,21]. Computer tomography is also used for studying the skeleton and bones, including the fine structure of the bones [22]. A combination of these, with more advanced technologies such as X-ray fluorescence [23], radiography, MRI, and immunohistochemistry, was introduced in studies which demonstrated the detailed anatomy of the stifle [14] and the brain [13]. Fused CT- and MRI-based reconstructive anatomy has a huge potential for visualizing the blood supply both in vivo [24] or postmortem [25] but their current use in veterinary medicine has many limitations [26]. It is not possible to execute such examinations in vivo on pachyderm mammals due to their size and no such postmortem analysis existed at the time of this study.

In this study, we aimed to find the supplying vessels of the digital cushion in elephants and to define the course of the digital arteries. Such results may be used in planning surgeries in affected regions to improve the recovery time and the overall safety of the procedure.

## 2. Materials and Methods

### 2.1. Specimen

The Somogy County Kaposi Mór Teaching Hospital, Dr. Baka József Diagnostic and Oncoradiological Centre received the left forelimb of a deceased 6-year-old female Asian elephant (Elephas maximus) from the Budapest Zoo and Botanical Garden for further investigations following an autopsy. The death was not related to any foot problems, the limb was in a good condition and kept in a +4 °C environment. It was cut at the level of the *antebrachium* with a bone saw. We cannulated the median artery with a PVC tube and secured it to prevent any leakage. The specimen was placed into a PVC carrying tube to prevent any movements during transportation between the CT and MR units.

### 2.2. Image Acquisition

The CT scan was performed at the Moritz Kaposi Teaching Hospital Dr. József Baka Diagnostic, Radiation Oncology, Research, and Teaching Center (Kaposvár, Hungary) with a Siemens Somatom Sensation Cardiac CT (multislice scanner, Siemens AG, Erlangen, Germany). We completed two separate scanning series. First, a native scan was performed from the middle of the antebrachium distally to the sole, with the following settings: transverse slices, caudal vision of image, 120 kV, 80 mAs, 0.6 mm slice thickness, 492 mm field of view (FOV) with isotropic voxels, and a total number of 2 × 1600 slices. After that the arteries of the forelimb were filled with 120 mL of Iomeron 400 solution (iomeprol, Bracco, Oxford, UK) via the cannula placed earlier in the median artery, and the scanning was repeated in the same position with the same settings. The reconstruction kernel was U40u. The series were saved in DICOM format.

After we completed the CT scan, the specimen was transferred to the MR unit (Siemens Avanto, 1.5 T, Siemens AG, Erlangen, Germany) avoiding any movements of the cadaver during transportation. 

**Figure 1:**
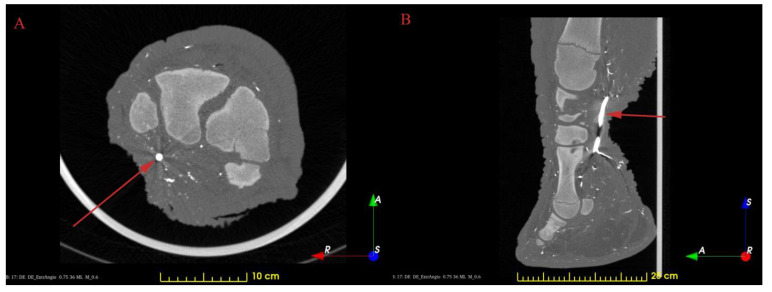
Contrast-filled CT images. The red arrows show the *a. mediana*. (**A**): horizontal plane; (**B**): sagittal plane.

Transverse, T1-weighted fast imaging low angle shot (FLASH) 3D gradient echo sequences were used for acquisition on 4 consecutive regions, covering the entire limb. Scan parameters were as follows: TR 8.03 ms, TE 4.78 ms, flip angle: 20, matrix: 320 × 320, FOV 450 mm, slice thickness 1.41 mm (isotropic voxels), NEX 1. The images were reconstructed without gaps and saved in DICOM format( Figure 1). 

### 2.3. Visualisation

After a thorough quality evaluation of the aforementioned data sets, the best series from each CT and MRI was chosen for the reconstruction of the bones and vasculature. The selected data sets were applied to the 3D Slicer program, version 4.10.2. A free and open-source software platform, 3D Slicer is utilizable for medical-image informatics, processing of images and visualisation with the aid of 3D models; its mainframe was developed with the support of the National Institutes of Health as well as with the help of a worldwide community of developers [27,28,29]. 

For the identification of the bones, we used the “Threshold” effect of the “Segment Editor” module as an automatic segmentation. The program recognizes structures of the same intensity range, stains them in the favoured colour, and generates a 3D model out of the given data. Afterwards, a careful, manual overview of each slice was performed to verify the borders and excess textures were deleted. After selection with the “Grow from seeds” effect, we were able to create a multicoloured 3D reconstruction of the limb.

The vessels were reconstructed with the “Subtract scalar volumes” module in the 3D Slicer. For this process, we needed to load a native sequence and one where the vessels were injected with the iodine contrast medium into the program. It then recognizes the difference between the two sequences (in this case the injected vessels) and is able to subtract them. 

We continued by using the “Segment editor” as before to obtain a 3D model for the forelimb’s vessels. A further step was to combine the reconstructed bones with the vessels to see their relationship with each other.

## 3. Results and Discussion

### 3.1. The Skeleton of the Forefoot

**Figure 2:**
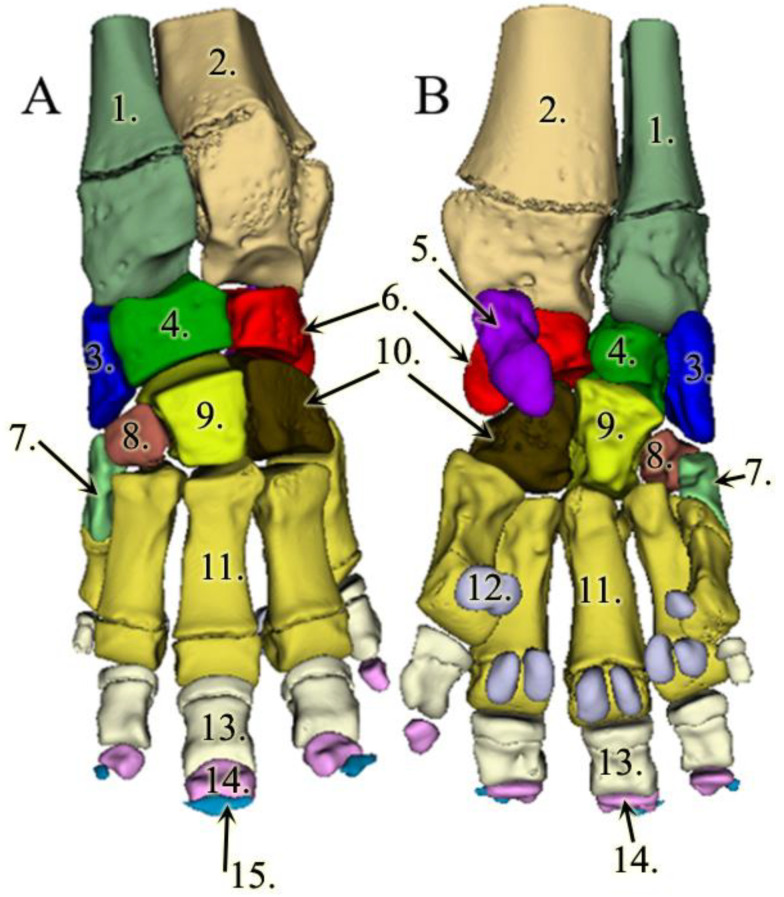
3D model of the left forelimb skeleton in an Asian elephant; (**A**): dorsal view; (**B**): palmar view. Antebrachium: 1: radius, 2: ulna, 3: os carpi radiale, 4: os carpi intermedium, 5: os carpi accessorium (only visible in palmar view), 6: os carpi ulnare, 7: os carpale I, 8: os carpale II, 9: os carpale III, 10: os carpale IV. 11: os metacarpale III. 12: ossa sesamoidea proximales (only visible in palmar view), 13: phalanx proximalis III, 14: phalanx media III., and 15: phalanx distalis III.

The reconstructions of the bones provided a unique, 3D view of the forelimb’s skeleton. The number of digits, toenails, and bones mostly correlate to the usual findings in the Asian elephant except that our specimen did not have a P-II phalange in D-I and the sesamoid bones of D-V were fused (Figure 2). 

**Figure 3:**
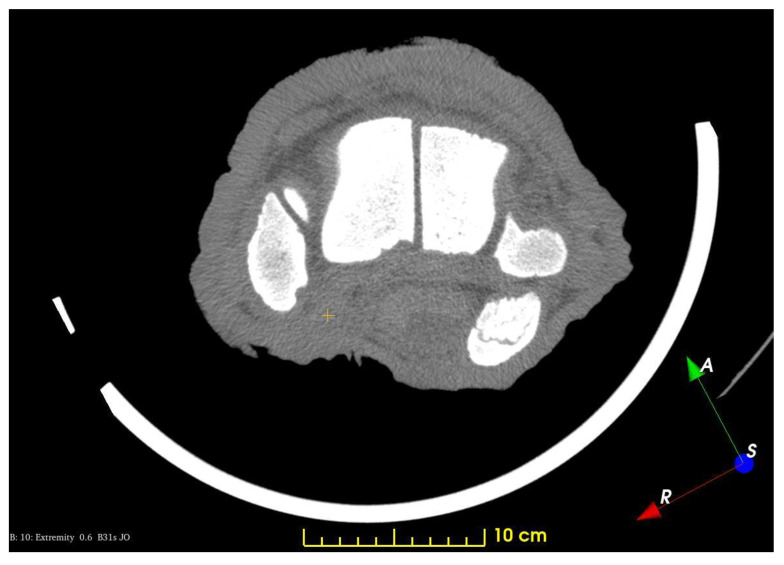
CT image, transverse view of the left forelimb at the distal carpal region. Yellow marker: prepollex.

The prepollex was completely unossified, and, consequently, the 3D Slicer could not identify it; therefore, it did not highlight it coevally with the bones. This is due to the image preparation, which was not appropriate for a definite identification of soft tissues and cartilaginous structures, but on the CT scans, a shadow at the correct location—at the distal end of C-I and the proximal end of MC-I—can be presumed to be the prepollex (Figure 3). These findings conform with previous investigations, where the predigits showed various appearances; they can stay cartilaginous, become partly ossified, or become completely ossified during an elephant’s life [8,10,11]. A similar study on a different, older specimen, with a more ossified prepollex, could potentially include its reconstruction. In our study, in order to obtain better separation between the soft-tissue elements, one would require advanced specimen preparation such as diffusible iodine-based contrast-enhanced computed tomography (DICECT, [30]), but that would have jeopardized the original goal of visualising the vessels. The results of manual tracing of the structures on the existing images would be questionable because the outlines are barely visible. Even in the case of such reconstruction, adding it to the current render would be misleading, as no other cartilages are presented. 

The distal growth plates of the antebrachium and crus were opened, as well as the proximal growth plates on the proximal phalanges (P-I) of the forefoot and the distal growth plates on the metacarpal bones. This is in line with the age of our subject, as these growth plates would only close by 9–10 years of age [31].

As the bones were reconstructed one by one, we were able to provide detailed images of each bone.

### 3.2. Results of the Vessel Reconstructions

To our knowledge, until today, no 3D models were made of the vessels of any elephant’s forefoot. In our model, the strong *a. mediana* is coursing down at the medial aspect of the antebrachium, more precisely on the palmar surface of the radius. At the carpal region, the median artery becomes the *a. metacarpea*. There are some small branches supplying the proximal row of the carpus. The additional blood supply for the carpus arises as recurrent branches from the middle dorsal part of the metacarpal bones. 

**Figure 4:**
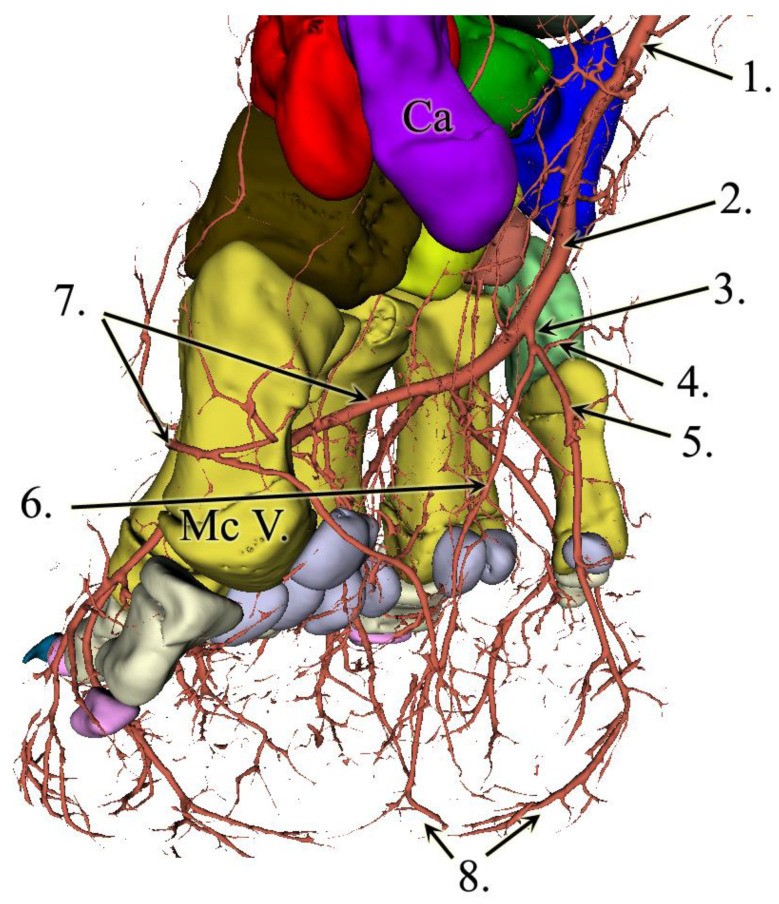
3D reconstruction of the forefoot vessels on the left limb. Latero-palmar view. 1: a. mediana, 2: a. metacarpea, 3: newly found vessel, 4: branch towards the first digit and prepollex, 5: palmar branch, 6: dorsal branch, 7: arcus palmaris profundus, 8: terminal sections of the dorsal and palmar branches headed towards each other in the digital cushion, and Ca: os carpi accessorium.

Just prior to the metacarpal artery becoming the deep palmar arch, we found a major vessel branching off in the palmar direction that was so far undescribed. The vessel has approximately the same diameter as any of the dorsal metacarpal arteries, it aims distally, and we were able to trace its branches up to the level of the sole. It remains close to the median plane of the foot, but it is slightly closer to the medial side. This vessel runs 8 mm after it emerges before it develops a trifurcation. The first branch of it turns medially, palmar to the first digit, and we assume that it supplies the prepollex as well. The other two branches remain in approximately the same vertical plane, although both are slightly bent in the lateral direction. One of them goes dorsally, the other palmarly, and both reach the sole approximately 7.5 cm apart from each other. Close to the sole, the vessels turn towards each other, getting very close, but we cannot confirm an anastomosis (Figure 4). 

**Figure 5:**
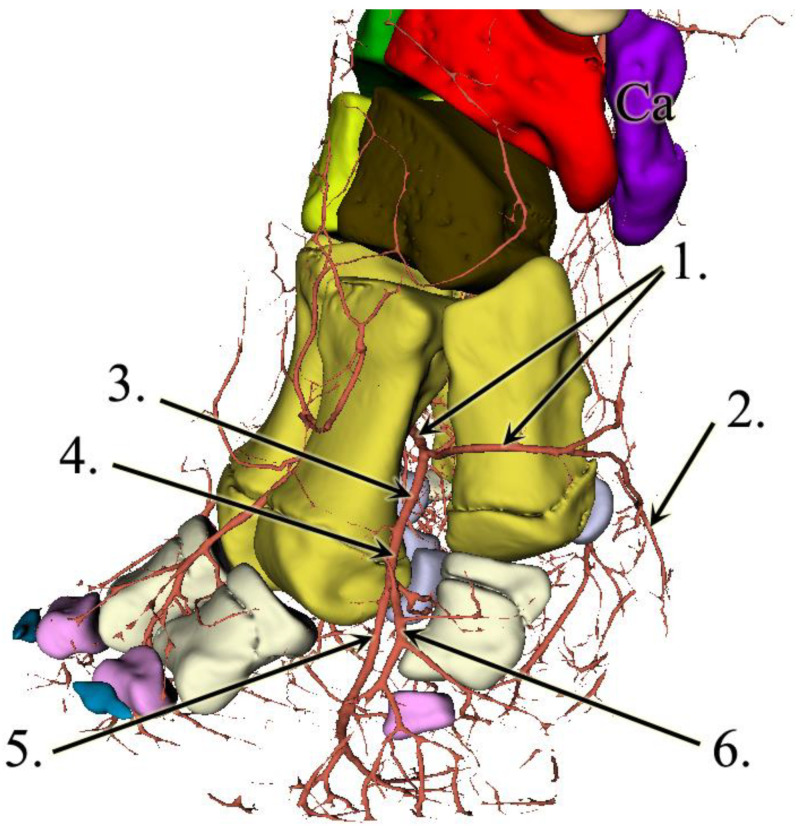
3D reconstruction of the forefoot vessels on the left limb. Lateral view. 1: arcus palmaris profundus, 2: lateral terminal branch at the dorso-lateral side of the digital cushion, 3: a. metacarpea dorsalis IV, 4: a. digitalis dorsalis communis IV, 5: a. digitalis dorsalis IV abaxialis, 6: a. digitalis dorsalis V axialis, and Ca: os carpi accessorium.

The deep palmar arch bends laterally and runs between D-IV and D-V before giving rise to the fourth common digital artery. The terminal section of the deep palmar arch goes around the fifth metacarpal bone from the dorsal direction, returns to the palmar side, and gets lost in the lateral side of the digital cushion (Figure 5). The metacarpal arteries that are branching off from the arch become common dorsal digital arteries as they go to the dorsal side, between the heads of the corresponding metacarpal bones. These common dorsal digital arteries bear axial and abaxial branches to the neighbouring phalanges. Branching off from the deep palmar arch, we also located relatively small vessels, the palmar digital arteries, that run on the palmar aspect of the metacarpus before dividing into two and getting lost at the height of the proximal sesamoid bones.

In the literature, there are no photos or detailed images for comparing the alignment of the vessels with our findings, only a few written explanations and schematic drawings. Based on these, we managed to find a new, so far undescribed, major vessel, which runs in the direction of the digital cushion. The blood supply for the digital cushion is well known in other ungulates such as ruminants and horses. In these species, the corresponding vessels exist usually as paired (abaxial/axial or lateral/medial) branches per limb, but discharge from more distal arteries [6,32]. The vessel in question also divided into two portions that descended to the digital cushion, but these coursed towards dorsal and palmar directions respectively, which could be due to the different pedal arrangement (semidigitigrade versus unguligrade) or the huge dorso-palmar extent of the cushion. Our findings are in line with a previous report [12] where a band of vessels and nerves are described in African elephants in the same plane but not discussed further. 

We did not find the branch of the metacarpal artery that goes to the first digit which was mentioned in the literature [3] but the medially directed branch of the trifurcation could be an alternatively flowing branch that also supplies the prepollex. Another, so far undescribed, feature of the vessels is that the *arcus palmaris profundus* runs between the fourth and fifth metacarpals and turns over to the dorsal aspect of the fifth metacarpal bone before returning to the palmar side, with its terminal section supplying the lateral wall of the digital cushion. We could not find the interosseus branch of the median artery, but it is possible that this branch departs the median artery at a position proximal to where the forelimb was cut; the contrast medium may also not have been able to enter this vessel because it was damaged during the cutting process.

## 4. Conclusions

The subject did not have any musculoskeletal disorders, and the CT and MR images also show regular anatomy on the forelimb; therefore, the results concerning the blood supply of the forefoot are most likely anatomically correct. Even though we only had the opportunity to examine one single specimen, the newly described vessel was the missing major branch in the blood supply of the digital cushion in Asian elephants. As this vessel runs in the direction of the digital cushion, we suggest the name “*a. tori digitalis communis”* until the trifurcation, whence for the three branches we suggest “*r. dorsalis*”, “*r. palmaris*”, and “*r. digitalis I*”. 

Additionally, we were able to find, visualize, and detail the course of the major arteries on the plantar aspect of the forefoot in the Asian elephant. We also created 3D models of the bones (Atlas S1) and vessels, which can be used for 3D printing as well, whilst the images and videos (Video S1) provide an immediate insight for education and surgical planning. Further reconstructions will be processed on these sequences to evaluate joint cavities and tendon sheets. For more fine anatomical details, such as innervation, standard dissection would have to be carried out.

## Data Availability

Data supporting reported results can be found 10.6084/m9.figshare.23054258.

## Supplementary Materials

The following supporting information can be downloaded at: https://www.mdpi.com/article/10.3390/ani13111789/s1, Atlas S1: Left carpal bones of the Asian elephant; Video S1: 3D animation of the Asian elephant’s forelimb blood supply.
