# Supplementary material for: 3D Reconstruction of the Blood Supply in an Elephant’s Forefoot Using Fused CT and MRI Sequences
Source: Animals (Basel). 2023 May 28;13(11):1789. doi: 10.3390/ani13111789 (PMC10252057; doi:10.3390/ani13111789)
Supplement: Supplementary file 1 [file animals-13-01789-s001.zip › Carpus atlas.pdf]

Left carpal bones of the Asian elephant

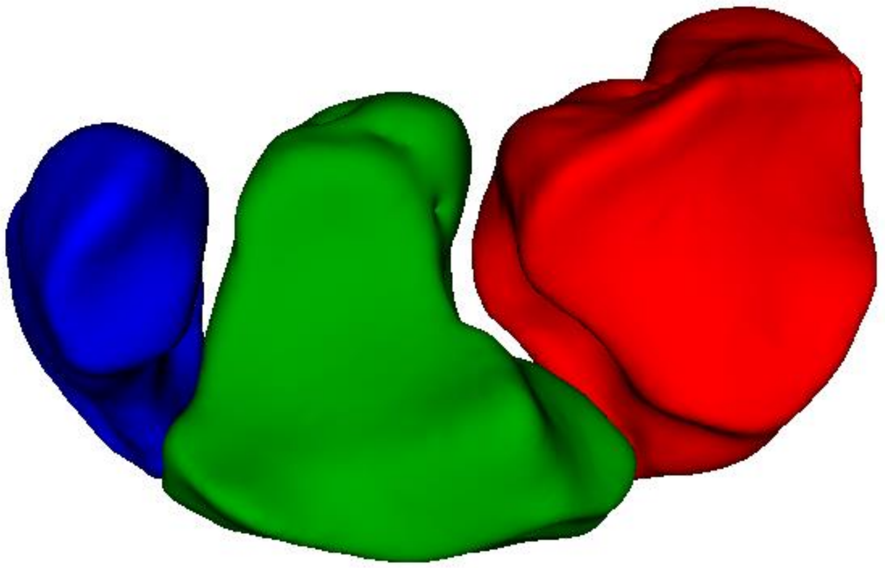

Proximal articular surfaces of the proximal carpal row (except for os carpi accessorium)

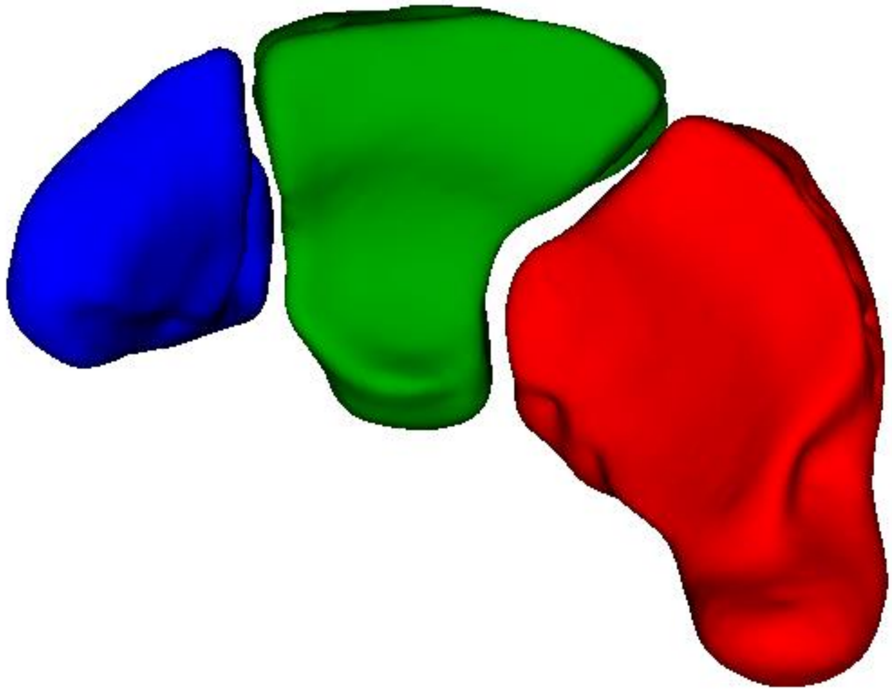

Blue: os carpi radiale, green: os carpi intermedium, red: os carpi ulnare

Distal articular surface of the proximal carpal row (except for os carpi accessorium)
Blue: os carpi radiale, green: os carpi intermedium, red: os carpi ulnare

A

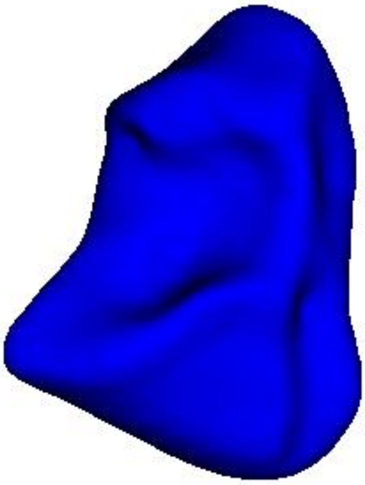

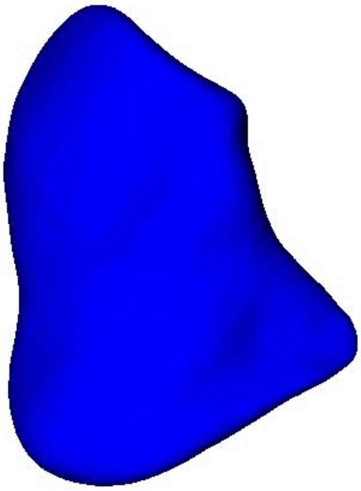

B

A: Lateral view of os carpi radiale (articular surface for intermedium)
B: medial view of os carpi radiale

A

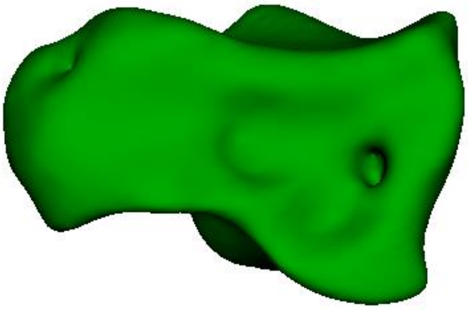

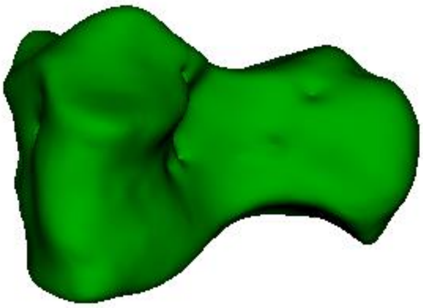

B

A: Medial view of os carpi intermedium– **B:** articular surface for os carpi radiale

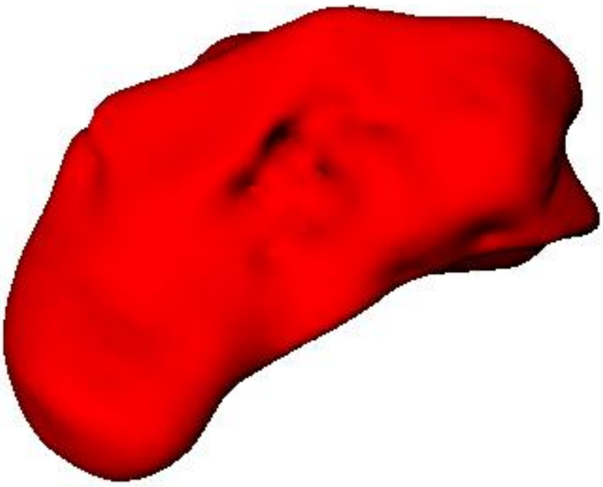

Palmar view of os carpi ulnare

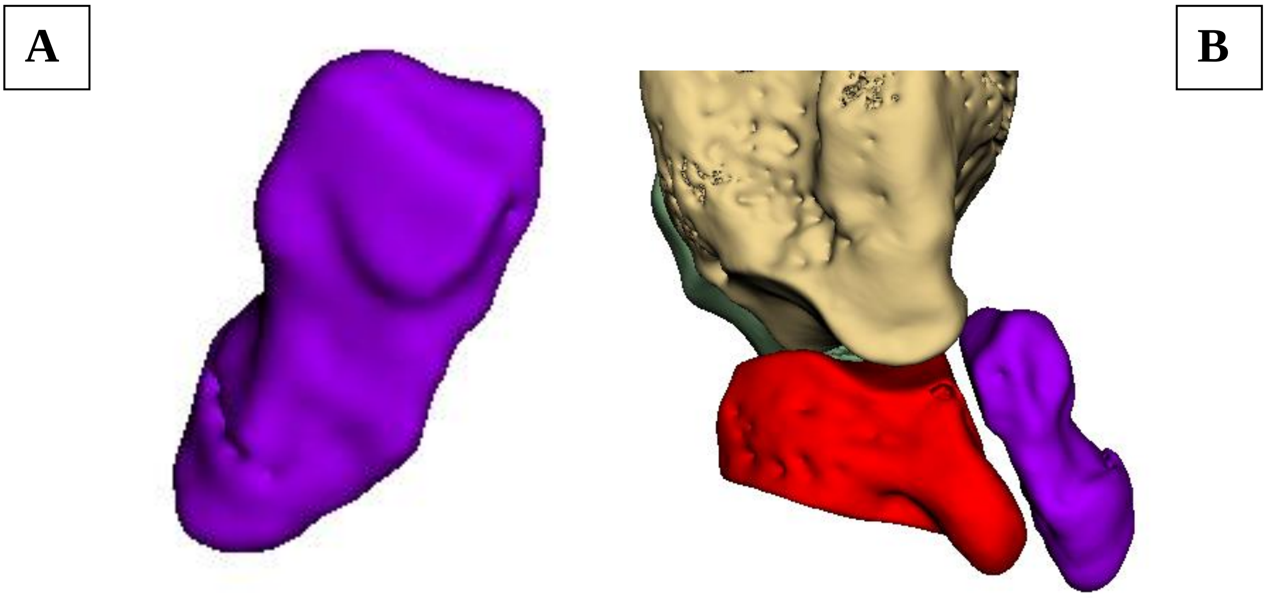

A: Dorso-medial view of os carpi accessorium, articular surface with ulna and os carpi ulnare
B: Lateral aspect of the os carpi accessorium (purple), the os carpi ulnare (red) and the ulna (beige)

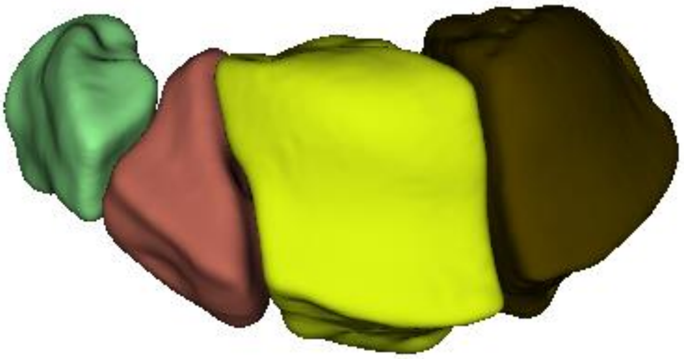

Proximal articular surfaces of the distal carpal row. Green: C-I, teak: C-II, yellow: C-III, brown: C-IV.

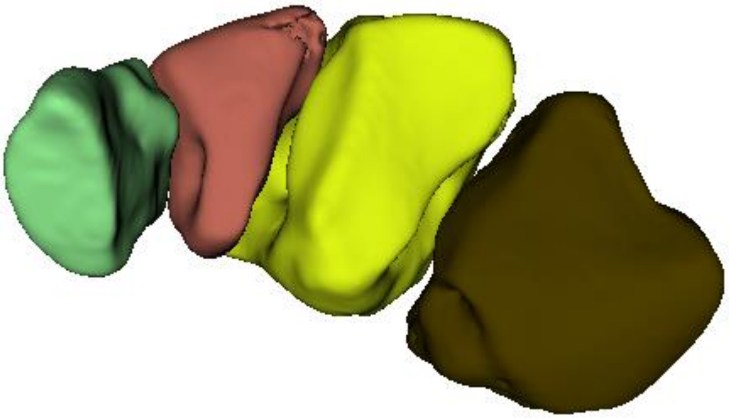

Distal articular surfaces of the distal carpal row. Green: C-I, teak: C-II, yellow: C-III, brown: C-IV.

A

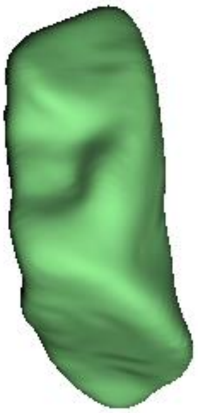

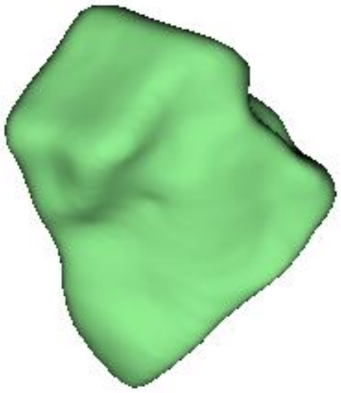

B

A: Palmar view of C-I
B: Lateral view, proximal articular surface for C-I

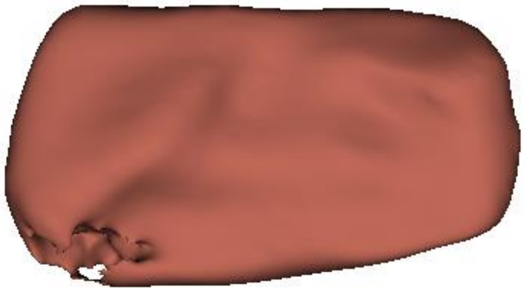

Lateral view of C-II, articular surface for C-III

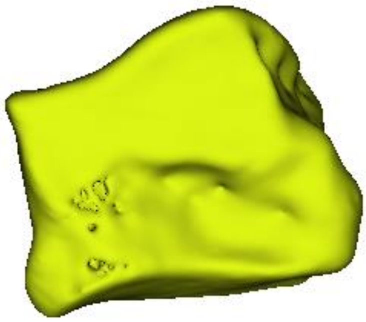

Lateral view of C-III, articular surface for C-IV
Lateral view of C-IV

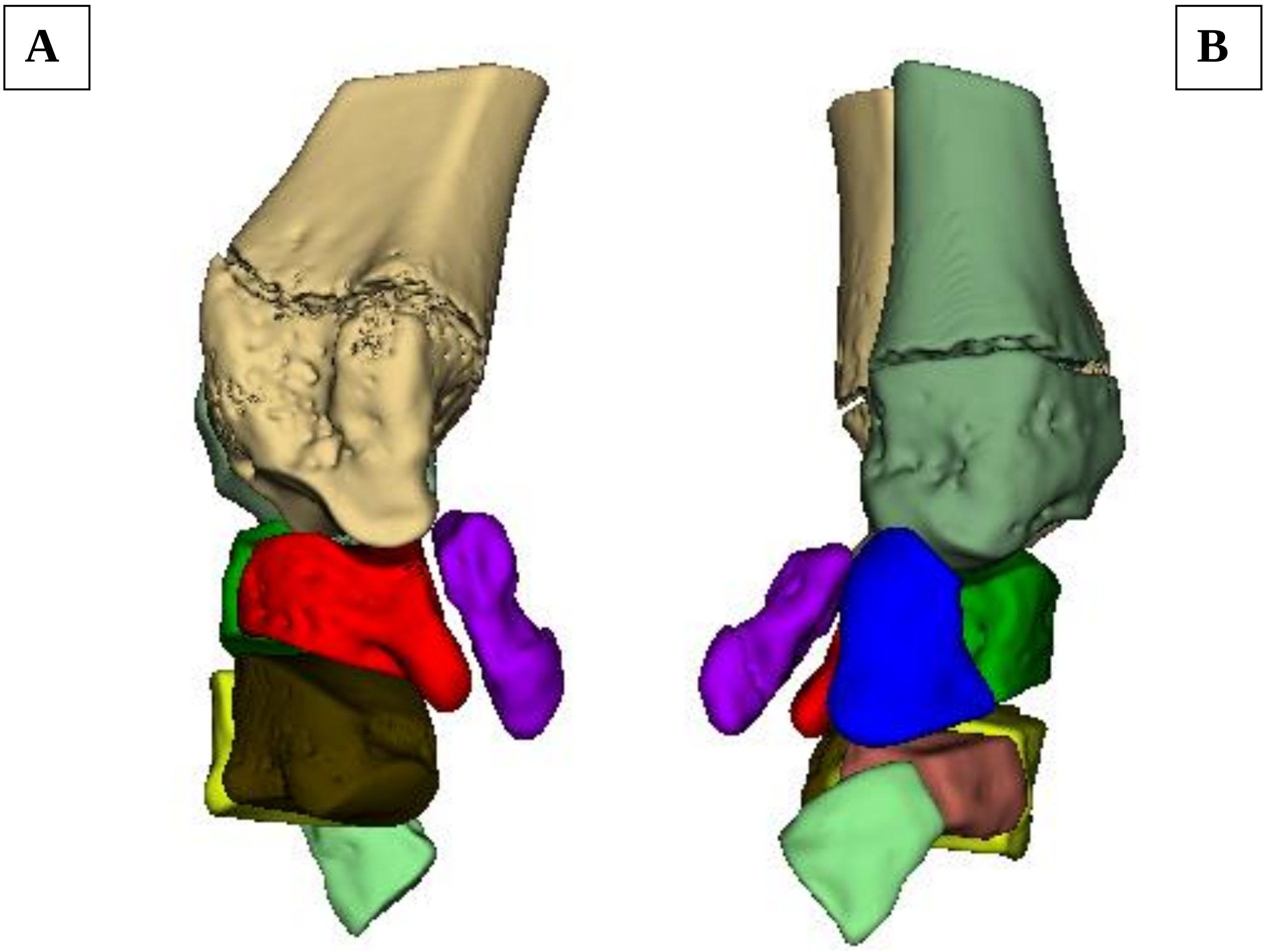

A: Lateral view of the carpal bones
B: Medial view of the carpal bones
