# Supplementary figures and images for: 3D Reconstruction of the Blood Supply in an Elephant’s Forefoot Using Fused CT and MRI Sequences
Source: Animals (Basel). 2023 May 28;13(11):1789. doi: 10.3390/ani13111789 (PMC10252057; doi:10.3390/ani13111789)

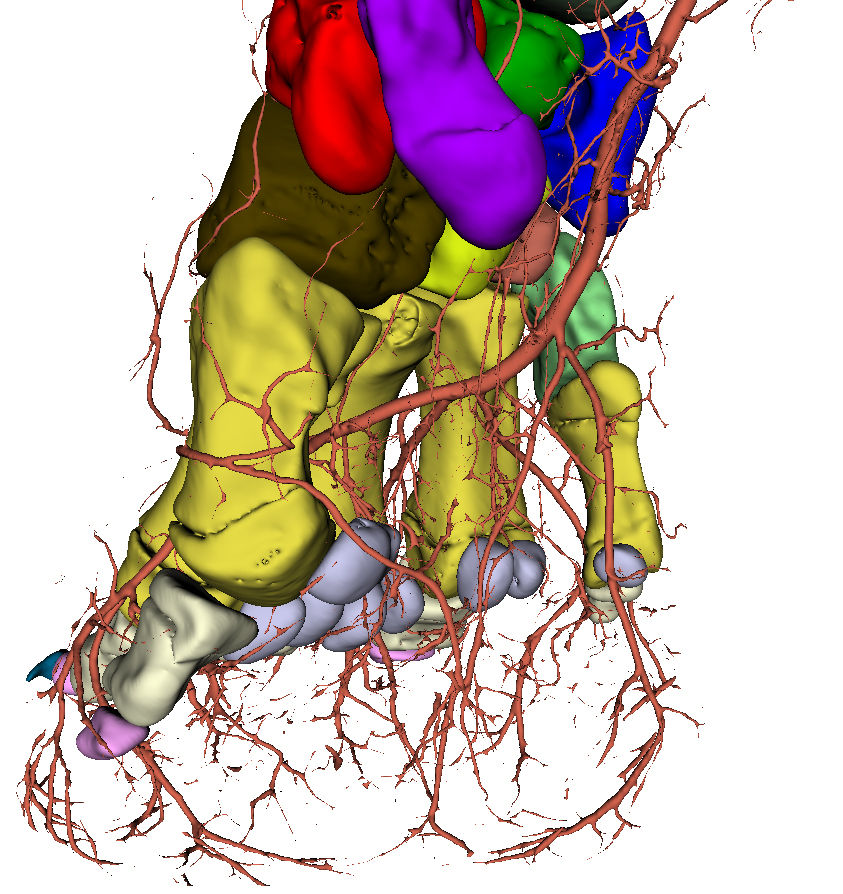

Supplement: Supplementary file 1 [file animals-13-01789-s001.zip › cover_img.jpg]
